# Depletion of globosides and isoglobosides fully reverts the morphologic phenotype of Fabry disease.

**DOI:** 10.1007/s00441-014-1922-9

**Published:** 2014-07-04

**Authors:** Stefan Porubsky, Richard Jennemann, Lorenz Lehmann, Hermann-Josef Gröne

**Affiliations:** 1 Department of Cellular and Molecular Pathology, German Cancer Research Center, Im Neuenheimer Feld 280, 69120 Heidelberg, Germany; 2 Institute of Pathology, University Medical Center Mannheim, University of Heidelberg, Theodor-Kutzer-Ufer 1-3, 68167 Mannheim, Germany; 3 Department of Cardiology, University of Heidelberg, Im Neuenheimer Feld 410, 69120 Heidelberg, Germany

**Keywords:** Fabry disease, αGalactosidase A, Electron microscopy, Lysosome, Globoside

## Abstract

Fabry disease is a monogenic X-linked lysosomal storage disease caused by α-galactosidase A (αGalA) deficiency. Enzyme replacement therapy through administration of the missing αGalA is currently the only accepted therapeutic option. However, this treatment is connected to high costs, has ill-defined indication criteria and its efficacy is controversially discussed. Our aim was to explore the possibility of a novel targeted substrate reduction therapy for Fabry disease. Owing to the fact that αGalA-deficient humans and mice accumulate the same glycosphingolipids (i.e. globosides, galabiosylceramide and isoglobosides), αGalA-deficient mice were crossed with mice deficient in enzymes synthesizing these classes of glycosphingolipids (i.e. globotrihexosylceramide and isoglobotrihexosylceramide synthase, respectively). Functional heart and kidney tests were performed together with an extensive biochemical analysis of urine and serum in aged mice. Lysosomal storage was assessed by thin layer chromatography and electron microscopy. We showed that depletion of globosides was sufficient to fully abolish the storage of glycosphingolipids in heart, kidney and liver and was paralleled by a complete restoration of lysosomal morphology in these organs. In contrast, in dorsal root ganglia, a depletion of both globosides and isoglobosides was necessary to fully counteract the lysosomal storage. The deficiency in globosides and/or isoglobosides did not cause any adverse effects. We conclude that substrate reduction therapy through inhibition of the synthesis of globosides and isoglobosides represents a valuable therapeutic option for Fabry disease, all the more as globosides and isoglobosides seem to be dispensable.

**Electronic supplementary material:**

The online version of this article (doi:10.1007/s00441-014-1922-9) contains supplementary material, which is available to authorized users.

## Introduction

**Fig. 1:**
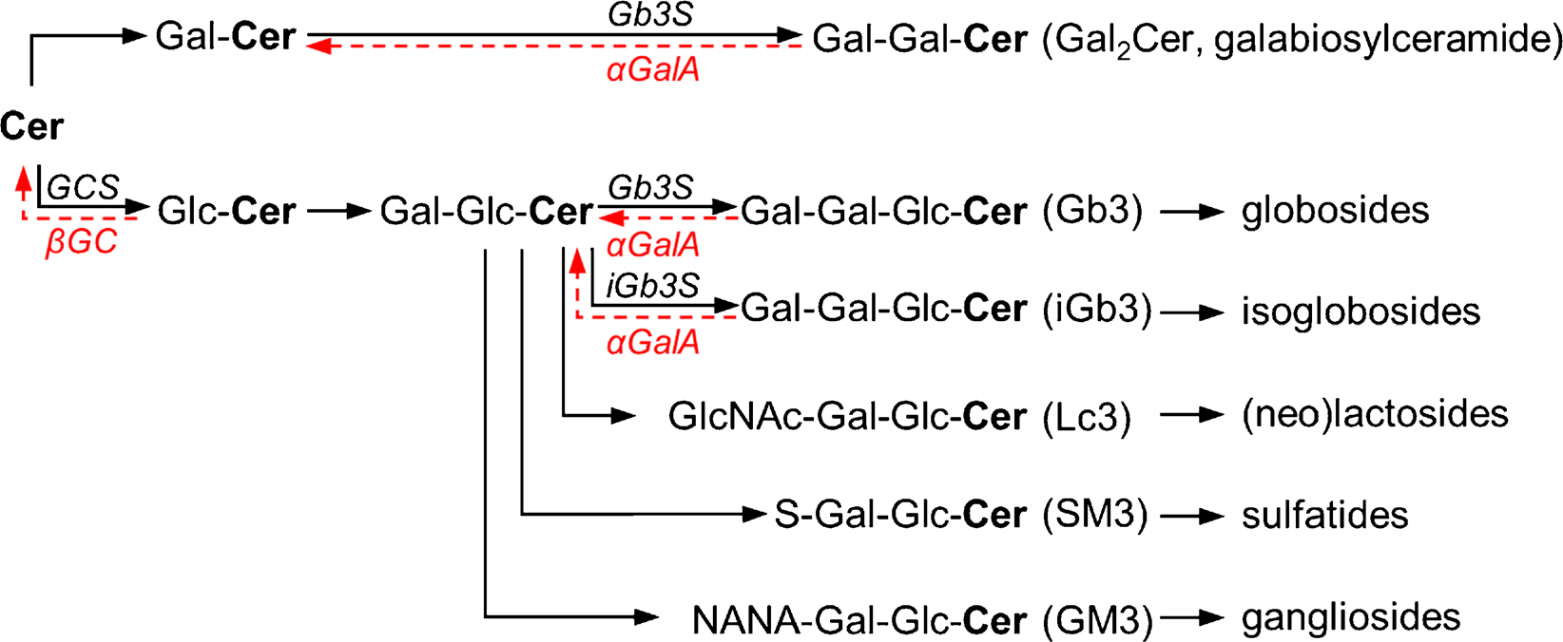
Glycosphingolipid metabolism. Relevant glycosphingolipids are depicted together with the corresponding synthesis (*black*) and degradation (*red*) enzymes. Depending on the first hexose moiety added to ceramide (*Cer*), either galactosylceramide (*GalCer*) or glucosylceramide (*GlcCer*) is formed. GlcCer is further processed to lactosylceramide (*LacCer*, i.e.* GalGlcCer*). Through the action of further enzymes on GalCer or LacCer, individual groups of GSL are produced, from which only the first member is shown for the sake of clarity. Globotrihexosylceramide synthase (*Gb3S*) is responsible for the synthesis of Gb3 and galabiosylceramide (*Gal*_*2*_ *Cer*, i.e.* GalGalCer*). Isoglobotrihexosylceramide synthase (*iGb3S*) synthesizes iGb3. α-Galactosidase A (*αGalA*), which catalyzes the degradation of Gb3, iGb3 and Gal_2_Cer, is deficient in Fabry disease. In contrast, patients with Gaucher disease suffer from GSL accumulation due to a deficiency in acid β-glucosidase (*βGC*).* GCS* glucosylceramide synthase;* GlcNAc* N-acetyl glucosamine;* NANA* N-acetylneuraminic acid;* S* sulfate

Fabry disease (Angiokeratoma corporis diffusum, OMIM 301500) was simultaneously described in 1898 by the German dermatologist Johannes Fabry and the British surgeon William Anderson (Fabry 1898; Anderson 1898). It is a monogenetic X-linked disorder characterized by different mutations of the α-galactosidase A (αGalA) gene including deletions, missense/nonsense, and frame-shift mutations or splice site defects (Brady et al. 1967; Topaloglu et al. 1999; Auray-Blais et al. 2008). The resulting decreased or absent activity of the enzyme αGalA leads to lysosomal accumulation of glycosphingolipids (GSL), the most prominent of them being globotrihexosylceramide (Gb3; Fig. 1) (Sweeley and Klionsky 1963; Hozumi et al. 1990).

Fabry disease is one of the most frequent storage disorders. The incidence of the classical phenotype is estimated as 1:40,000. Recent studies, also including late-onset (non-classical) phenotypes, have reported an incidence between 1:3,100 to 1:4,600 newborns (Spada et al. 2006; Hwu et al. 2009). Random inactivation of one of the X-chromosomes in females and mutations with partially preserved enzyme activity lead to atypical or attenuated disease manifestations.

In male patients with the classical phenotype, clinical symptoms usually begin in childhood and include characteristic skin lesions (angiokeratomas), acroparesthesias, corneal opacity, and hypohidrosis. The kidney involvement manifests as proteinuria and declining kidney function which inevitably progresses to end-stage kidney disease. Cardiac manifestations include left ventricular hypertrophy, conduction abnormalities, and coronary artery disease with ensuing congestive heart failure, arrhythmias, and myocardial infarction, respectively. Transient ischemic attacks, early strokes, white matter lesions, vertigo, and hearing loss belong to further prominent, however unspecific, symptoms (reviewed in Desnick et al. 2003; Clarke 2007; Schiffmann 2009).

Currently, the only accepted treatment for Fabry disease is enzyme replacement therapy (ERT) with one of the two preparations: Agalsidase-alfa (Replagal^®^; Shire HGT) or agalsidase-beta (Fabrazyme^®^; Genzyme) (Brady 2006; Schiffmann 2009).

Although ERT is generally well tolerated and has been shown to reduce the tissue and plasma Gb3 concentration in Fabry patients, no consistent evidence of clinical efficacy could be provided (Sheppard et al. 2010; Pisani et al. 2012; Rombach et al. 2013a; El Dib et al. 2013; Tøndel et al. 2013). Due to the low prevalence of the disease and variable definitions of end-points in the available studies, therapy indications are still ill-defined—a non-negligible aspect with regard to the substantial costs of the aforementioned ERT drugs (Rombach et al. 2013b). Moreover, ERT is linked to several problems such as insufficient tissue availability (e.g., due to the blood–brain barrier) or formation of antibodies against αGalA which may have neutralizing effects or lead to infusion-associated reactions (Ohashi et al. 2008; Deegan 2012; Rombach et al. 2012; Wilcox et al. 2012).

As a monogenic disorder, Fabry disease might also be amenable to gene therapy. Although this approach has been repeatedly tested in mice, the achievement of a safe delivery of the nucleic acid to all target cells and a sustained expression of the enzyme are still the major hurdles of such therapy in humans (Jung et al. 2001; Przybylska et al. 2004; Yoshimitsu et al. 2006; Choi et al. 2010).

A different approach in the treatment of storage disorders is represented by substrate reduction therapy (SRT). In contrast to Fabry patients, individuals with Gaucher disease suffer from lysosomal storage of all GlcCer-derived GSL (Fig. 1). For the treatment of Gaucher patients, an SRT has been established and is based on the inhibition of GlcCer synthase (GCS) (Zimran 2011). This approach is also being considered for Fabry disease (Abe et al. 2000; Platt et al. 2003; Marshall et al. 2010). However, GCS inhibition depletes more GSL groups than would be needed to interfere with the storage caused by αGalA deficiency in Fabry patients (Fig. 1). As GlcCer-derived GSL play an indispensable role in numerous biological processes such as embryogenesis, central and peripheral nervous function, or epidermal skin barrier (Jennemann and Gröne 2013), their depletion may under certain circumstances be accompanied by adverse effects (Hollak et al. 2009; Machaczka et al. 2012).

We hypothesized that, for Fabry disease, an SRT by a targeted depletion of globosides and/or isoglobosides would suffice to counteract the lysosomal storage phenotype without affecting the synthesis of other GlcCer-based GSL and thus potentially lead to fewer side-effects. Our results demonstrate in vivo that interfering with the synthesis of globosides and isoglobosides fully reverted the Fabry phenotype. Therefore, an SRT by inhibition of enzymes synthesizing globosides and isoglobosides represents a valuable therapeutic option for Fabry disease and should be considered in future research.

## Materials and methods

### Mice

Mice deficient for Gb3S (EC 2.4.1.228, A4galt^tm1.1Poru^) and iGb3S (EC 2.4.1.87, A3galt2^tm1.1Hjg^) were generated by our group and backcrossed for more than 10 generations to the C57BL/6 genetic background (Porubsky et al. 2007, 2012). Mice deficient for αGalA (EC 3.2.1.22, Gla^tm1Kul^) were provided by Ashok Kulkarni (Ohshima et al. 1997). All strains were housed under specific pathogen-free conditions and kept under a 12/12 h light/dark cycle with access to water and regular chow ad libitum.

Urine was collected using metabolic cages with water access ad libitum. Analysis of plasma and urine samples was performed on an Hitachi 9-17E analyser (Hitachi, Frankfurt, Germany). Creatinine clearance was calculated as U_Cr_xV_24_/(P_Cr_x24x60), where U_Cr_ and P_Cr_ are creatinine concentrations in urine and plasma, respectively, and V_24_ is the 24-h urine volume. Peripheral blood, spleen, and thymus were analyzed by flow cytometry as detailed in Porubsky et al. (2007, 2012).

### Transthoracic echocardiography

Non-invasive transthoracic echocardiography was performed in a modified setting as previously described (Malekar et al. 2010). In brief, a two-dimensional parasternal short axis view and M-mode tracings of the left ventricle were obtained with a Sonos 5500 echocardiogram (Philips, Andover, MA, USA) using a S12 transducer (12 MHz). M-mode tracings were used to measure left ventricular internal diameter (LVID) as the largest anteroposterior diameter in either diastole (LVIDd) or systole (LVIDs) and averaged from at least three consecutive cardiac cycles. Left ventricular fractional shortening was calculated as (LVIDd-LVIDs)/LVIDd and expressed as a percentage. The heart rate was calculated based on the M-mode tracings.

### Isolation and analysis of GSL

Freshly harvested organs were frozen in liquid nitrogen and lyophilized. Tissues were powdered and dry weight was determined. Extraction of GSL from organs and the subsequent analysis by thin layer chromatography (TLC) were performed as described in detail in Porubsky et al. (2012); briefly, GSL were eluted from organs using CHCl_3_/CH_3_OH/H_2_O. In order to eliminate phospholipids and triglycerides, crude extracts were treated with 1 ml 0.1 M KOH in CH_3_OH at 50 °C for 4 h. After neutralization with acetic acid and evaporation of CH_3_OH, potassium acetate was removed from lipids via reversed phase column chromatography (RP18). Neutral and acidic (sialic acid-containing) GSL were separated by ion exchange chromatography.

For TLC, an amount corresponding to 2 mg dry organ weight was loaded. Running solvent was CHCl_3_/CH_3_OH/H_2_O (62.5:30:6, v/v/v) for neutral GSL and CHCl_3_/CH_3_OH/ 0.2% CaCl_2_ in H_2_O (60:35:8, v/v/v) for acidic GSL. To visualize GSL, the TLC plate was sprayed with 0.2 % orcinol in 10 % sulfuric acid and incubated 10 min at 120 °C.

### Histology and immunohistochemistry

Tissue samples were fixed in 4 % phosphate buffered formaldehyde, paraffin-embedded, and cut at 4 μm. The cuts were subjected to routine staining procedures including hematoxylin and eosin stain (HE), periodic acid-Schiff (PAS), and Goldner stain. For Gb3-immunohistochemistry, cryosections were fixed with formaldehyde and probed with polyclonal chicken anti-Gb3 antibody JM06/298-1 (Betz et al. 2011) followed by alkaline phosphatase-conjugated polyclonal donkey anti-IgY secondary antibody (Jackson ImmunoResearch Europe, Suffolk, UK). Slides were scanned by Mirax Scan (Carl Zeiss, Germany). Pictures were exported using the Panoramic Viewer (3DHISTECH, Budapest, Hungary).

### Electron microscopy

Organs were fixed in Karnovsky’s glutaraldehyde (2 % paraformaldehyde, 2.5 % glutaraldehyde, 0.2 M cacodylate buffer pH 7.4) and embedded in araldite (Serva, Heidelberg, Germany). Ultrathin sections were stained with lead citrate and uranyl acetate. Photographs were taken on an electron microscope (EM 910; Carl Zeiss).

### Statistical analysis

Unpaired two-tailed Student’s *t* test was performed to compare datasets. Differences were considered significant if *p* < 0.05.

## Results

### Characterization of the gene deficient mice strains

Although αGalA-deficient mice accumulate the same GSL as humans, only a weak or non-functional organ impairment has been previously reported in αGalA-deficient mice (Ohshima et al. 1999; Yoshimitsu et al. 2006; Noben-Trauth et al. 2007; Marshall et al. 2010). Because all mice strains described in this study were backcrossed for more than 10 generations to the C57BL/6 genetic background and were strictly kept under identical conditions, we first sought to test whether, under these circumstances, any functional alterations would be detectable in αGalA-deficient mice in comparison to WT mice.

**Fig. 2:**
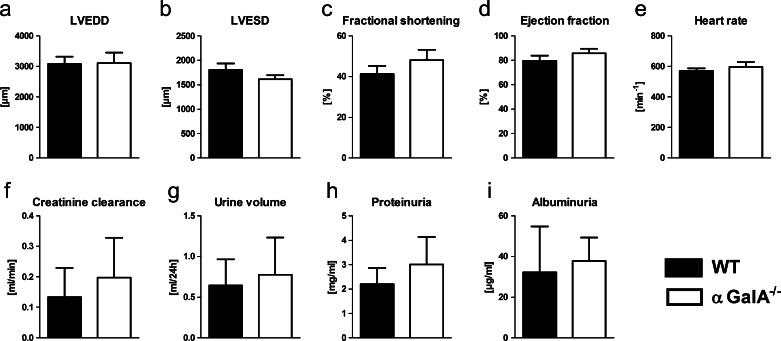
Functional analysis of the heart and kidney. In aged (1.5-year-old) α*GalA* ^*−/−*^ female mice, heart and kidney function were measured. **a**–**e** The heart function was tested using transthoracic echocardiography. No significant difference of the left-ventricular end-diastolic diameter (LVEDD, **a**), left-ventricular end-systolic diameter (LVESD, **b**), left ventricular fractional shortening (**c**) and ejection fraction (**d**) could be observed between α*GalA* ^*−/−*^ and WT mice. Similarly, no difference in the heart rate could be demonstrated between αGalA-deficient and WT mice (**e**). *Bars* represent means, and *whiskers* standard deviations, *n* = 5 and 8 for WT and α*GalA* ^*−/−*^ mice, respectively. **f**–**i** The kidney function was tested based on serum creatinine and analysis of 24-h urine. No differences in the creatinine clearance (**f**), urine volume (**g**), proteinuria (**h**), and albuminuria (**i**) could be demonstrated between α*GalA* ^*−/−*^ and WT mice. *Bars* represent means, and *whiskers* standard deviations, *n* = 9

Transthoracic echocardiography did not reveal any significant difference of the left-ventricular end-diastolic diameter (LVEDD), left-ventricular end-systolic diameter (LVESD), left ventricular fractional shortening (LVFS), and ejection fraction in aged (1.5-year-old) αGalA-deficient mice as compared to WT mice (Fig. 2a–d). Similarly, the heart rate was not altered in αGalA-deficient mice (Fig. 2e). Assessment of the kidney function using metabolic cages did not show any significant changes in creatinine clearance, urine volume, proteinuria, and albuminuria in αGalA-deficient mice in comparison to WT mice (Fig. 2f–i).

As further detailed in the Table 1, αGalA deficiency did not impact body and organ weights; the only exception being the kidney weight which was significantly increased in α*GalA* ^*−/−*^ females as compared to WT, α*GalA* ^*−/−*^ */Gb3S* ^*−/−*^, and *Gb3S* ^*−/−*^ female mice. Similarly, among all biochemical parameters tested in serum and urine, an increased cholesterol level could only be observed in α*GalA* ^*–/0*^ male mice as compared to WT, α*GalA* ^*–/0*^ */Gb3S* ^*−/−*^, and *Gb3S* ^*−/−*^ male mice (Table 1).

**Table 1:** Anatomical and biochemical parameters

**Females**	WT	αGalA^−/−^	αGalA^−/−^/Gb3S^−/−^	Gb3S^−/−^
Body weight (g)	25.8 ± 4.1	25.9 ± 2.2	27.4 ± 3.2	26.8 ± 3.3
Lungs (g)	0.160 ± 0.030	0.171 ± 0.024	0.161 ± 0.014	0.156 ± 0.014
Heart (g)	0.164 ± 0.026	0.176 ± 0.017	0.176 ± 0.018	0.184 ± 0.036
Liver (g)	1.203 ± 0.153	1.496 ± 0.455	1.312 ± 0.131	1.229 ± 0.182
Spleen (g)	0.120 ± 0.032	0.129 ± 0.051	0.111 ± 0.030	0.096 ± 0.035
Kidneys (g)	0.287 ± 0.031	**0**.**331** ± **0**.028 *	0.305 ± 0.024	0.283 ± 0.032
S-Creatinine (mg/dl)	0.11 ± 0.05	0.10 ± 0.02	0.09 ± 0.01	0.09 ± 0.01
S-Urea (mg/dl)	41.8 ± 17.5	47.6 ± 10.8	51.7 ± 15.1	50.4 ± 10.4
S-Protein (mg/ml)	59.3 ± 10.2	52.6 ± 7.8	55.8 ± 5.8	54.1 ± 2.6
S-Glucose (mg/dl)	128.8 ± 25.8	124.5 ± 23.3	110.6 ± 15.4	116.2 ± 22.5
S-Cholesterol (mg/dl)	98.7 ± 14.3	95.4 ± 11.1	101.5 ± 17.7	89.1 ± 12.6
S-TAG (mg/dl)	74.2 ± 18.5	81.0 ± 14.2	68.1 ± 16.3	67.1 ± 16.6
U-Creatinine (mg/dl)	31.9 ± 11.3	33.8 ± 12.7	28.8 ± 12.9	37.7 ± 23.7
U-Urea (mg/dl)	3,738 ± 1,411	5,290 ± 2,167	3,903 ± 1,516	5,419 ± 2,788
U-Glucose (mg/dl)	8.00 ± 8.44	13.67 ± 7.60	9.80 ± 9.61	15.43 ± 13.94
U-Na^+^ (mmol/l)	66.3 ± 60.1	95.1 ± 58.0	59.3 ± 34.2	73.3 ± 44.2
U-K^+^ (mmol/l)	137.3 ± 92.3	176.8 ± 104.5	136.5 ± 71.6	161.5 ± 96.3
U-Ca^++^ (mmol/l)	3.17 ± 1.82	3.81 ± 1.17	3.12 ± 1.49	2.66 ± 1.53
U-Phosphate (mmol/l)	58.7 ± 20.5	89.7 ± 50.2	65.8 ± 38.3	71.5 ± 47.3
**Males**	WT	αGalA^−/0^	αGalA^−/0^/Gb3S^−/−^	Gb3S^−/−^
Body weight (g)	34.0±6.6	37.3±5.0	38.5±4.9	35.6±5.7
Lungs (g)	0.164 ± 0.018	0.170 ± 0.020	0.167 ± 0.019	0.160 ± 0.027
Heart (g)	0.223 ± 0.038	0.205 ± 0.039	0.244 ± 0.054	0.231 ± 0.045
Liver (g)	1.724 ± 0.379	1.532 ± 0.230	1.567 ± 0.309	1.494 ± 0.224
Spleen (g)	0.094 ± 0.058	0.094 ± 0.015	0.082 ± 0.023	0.080 ± 0.039
Kidneys (g)	0.400 ± 0.085	0.396 ± 0.035	0.366 ± 0.033	0.372 ± 0.045
S-Creatinine (mg/dl)	0.14 ± 0.23	0.09 ± 0.02	0.10 ± 0.02	0.09 ± 0.02
S-Urea (mg/dl)	50.5 ± 14.6	46.3 ± 7.4	56.3 ± 22.0	60.2 ± 24.6
S-Protein (mg/ml)	58.1 ± 5.7	59.2 ± 4.5	61.1 ± 7.2	59.8 ± 5.8
S-Glucose (mg/dl)	125.4 ± 62.0	143.9 ± 56.9	120.3 ± 39.6	99.0 ± 27.4
S-Cholesterol (mg/dl)	116.8 ± 26.6	**167**.**9** ± **27**.**1 #**	123.6 ± 22.3	113.1 ± 16.2
S-TAG (mg/dl)	96.1 ± 25.3	105.7 ± 18.1	89.9 ± 26.6	92.9 ± 31.7
U-Creatinine (mg/dl)	33.1 ± 12.1	34.2 ± 21.2	23.9 ± 9.5	31.6 ± 10.9
U-Urea (mg/dl)	6,068 ± 2,803	4,632 ± 1,677	3,3662 ± 1,318	4,161 ± 992
U-Glucose (mg/dl)	18.64 ± 23.20	13.40 ± 8.95	6.91 ± 8.61	13.77 ± 8.11
U-Na^+^ (mmol/l)	125.3 ± 103.2	90.2 ± 45.3	81.0 ± 34.8	90.5 ± 51.5
U-K^+^ (mmol/l)	182.9 ± 125.5	132.8 ± 54.1	144.4 ± 62.7	135.6 ± 54.3
U-Ca^++^ (mmol/l)	2.65 ± 1.75	3.69 ± 2.11	2.56 ± 0.83	3.49 ± 2.06
U-Phosphate (mmol/l)	90.5 ± 52.0	68.6 ± 30.4	60.3 ± 27.1	58.1 ± 23.4

*αGalA^−/−^ females had a significantly higher (*p* < 0.05) kidney weight than WT, αGalA^−/−^/Gb3S^−/−^ and Gb3S^−/−^ females

#αGalA^–/0^ males had significantly higher (*p* < 0.001) serum cholesterol levels than WT, αGalA^–/0^/Gb3S^−/−^ and Gb3S^−/−^ males

*S* serum; *TAG* triacylglycerol; *U* urine

Values represent mean±SD; n=9-13 mice per group

Mice with deficiency for globotrihexosylceramide synthase (Gb3S) and isoglobotrihexosylceramide synthase (iGb3S) have been previously reported, and the deficiency in globosides and/or isoglobosides did not impact either organ histology or any physiological function so far tested (Electronic Supplementary Material, Fig. S1; Porubsky et al. 2007, 2012).

Due to the absence of a functional organ impairment in αGalA-deficient mice with a pure genetic background, we based all further investigations on biochemical and ultrastructural analyses.

### Biochemical analysis of GSL stored in αGalA-deficient mice

In αGalA-deficient humans and mice, Gb3 has been implicated as the most prominent GSL stored (Fig. 1). In order to test this in a genetic approach, αGalA-deficient mice were crossed with *Gb3S* ^*−/−*^ mice (α*GalA* ^*−/−*^ */Gb3S* ^*−/−*^) and tissue GSL were extracted. The analysis was focused on kidney, liver, and heart as these organs have been previously shown to have a prominent GSL accumulation in humans and mice (Hozumi et al. 1990; Ohshima et al. 1999).

**Fig. 3:**
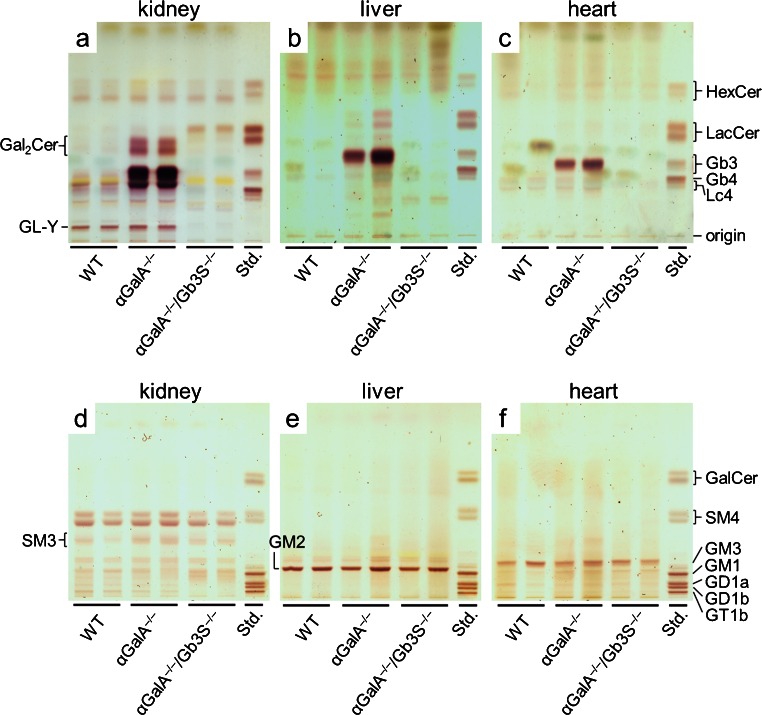
Analysis of GSL from selected organs. Neutral (**a**–**c**) and acidic (**d**–**f**) GSL extracted from kidneys, livers, and hearts of WT, α*GalA* ^*−/−*^, and α*GalA* ^*−/−*^ */Gb3S* ^*−/−*^ mice were analyzed by TLC. In all three organs, αGalA deficiency resulted in accumulation of a neutral GSL which ran at the height of Gb3 and was absent in the α*GalA* ^*−/−*^ */Gb3S* ^*−/−*^ double knockout thus confirming its identity as Gb3 (**a**–**c**). Accumulation of galabiosylceramide (*Gal*_*2*_ *Cer*) contributed to the storage in kidneys of α*GalA* ^*−/−*^ mice (**a**), but was—in agreement with being a product of Gb3S—absent in α*GalA* ^*−/−*^ */Gb3S* ^*−/−*^ kidneys. Gb3S deficiency resulted in a slight accumulation of its substrate lactosylceramide (*LacCer*) in kidneys (**a**). Among the acidic GSL (**d**—**f**), no accumulation was observed in α*GalA* ^*−/−*^ mice. Some GSL are represented by multiple bands due to their heterogeneous composition of fatty acids resulting in different running properties. Orcinol staining. *Std.* GSL standard

TLC analysis of neutral GSL extracted from αGalA-deficient mice showed that, in kidney, liver, and heart, the most prominent accumulating GSL ran at the height of Gb3 (Fig. 3a–c). In addition, in kidneys of αGal-deficient mice, galabiosylceramide, another product of Gb3S, accumulated. In the α*GalA* ^*−/−*^ */Gb3S* ^*−/−*^ double knockout-mice, the deficiency in Gb3S could fully abolish the GSL accumulation as detected by TLC (Fig. 3a–c). As a consequence of the Gb3S deficiency, a slight increase in its substrate LacCer could be seen in kidneys of α*GalA* ^*−/−*^ */Gb3S* ^*−/−*^ mice (Fig. 3a–c). However, this did not led to any compensatory increase in other LacCer-derived GSL (Figs. 1, 3). No accumulation of acidic GSL occurred in αGalA-deficient mice (Fig. 3d–f).

### Impact of Gb3S deficiency on the storage phenotype in αGalA-deficient mice

**Fig. 4:**
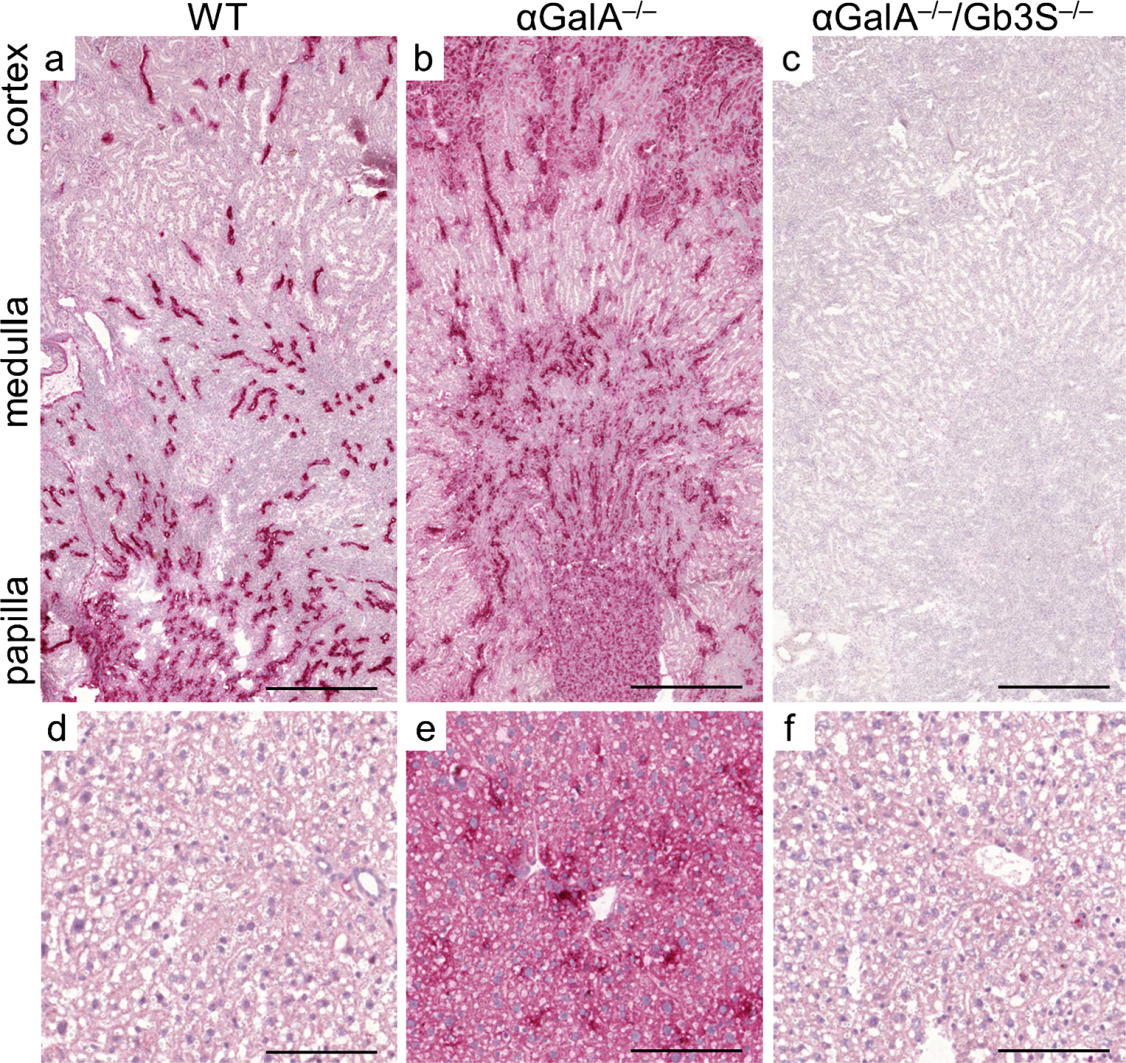
Immunohistochemical analysis of the Gb3 storage. Immunohistochemistry was performed using Gb3-antibodies in kidneys (**a**–**c**) and livers (**d**–**f**) of WT, α*GalA* ^*−/−*^, and α*GalA* ^*−/−*^ */Gb3S* ^*−/−*^ mice. Gb3 was prominently present in renal collecting ducts of WT mice but also emerged in other nephron segments in αGalA deficiency. Similarly, in the liver, Gb3 also accumulated in α*GalA* ^*−/−*^ mice. Gb3S deficiency abolished this storage in α*GalA* ^*−/−*^ */Gb3S* ^*−/−*^ mice. *Scale bars* (**a**–**c**) 0.5 mm, (**d**–**f**) 0.1 mm

To corroborate the TLC findings with histology, immunohistochemistry with anti-Gb3 antibodies was performed on kidney and liver tissue. In WT kidneys, a strong Gb3 expression could be observed in collecting ducts (Fig. 4a). In αGalA-deficient mice, the Gb3 synthesis became apparent due to the absence of its catabolism also in other nephron segments (Fig. 4b). In contrast, glomerular cells stained negative for Gb3 in WT mice as well as in αGalA-deficient mice (data not shown). In congruence with the TLC analysis, livers showed no Gb3 expression upon immunohistochemical investigation in WT mice but its accumulation was present in αGalA-deficient animals (Fig. 4d, e). Gb3S deficiency abolished the accumulation of globosides observed in organs of αGalA-deficient mice (Fig. 4c, f).

**Fig. 5:**
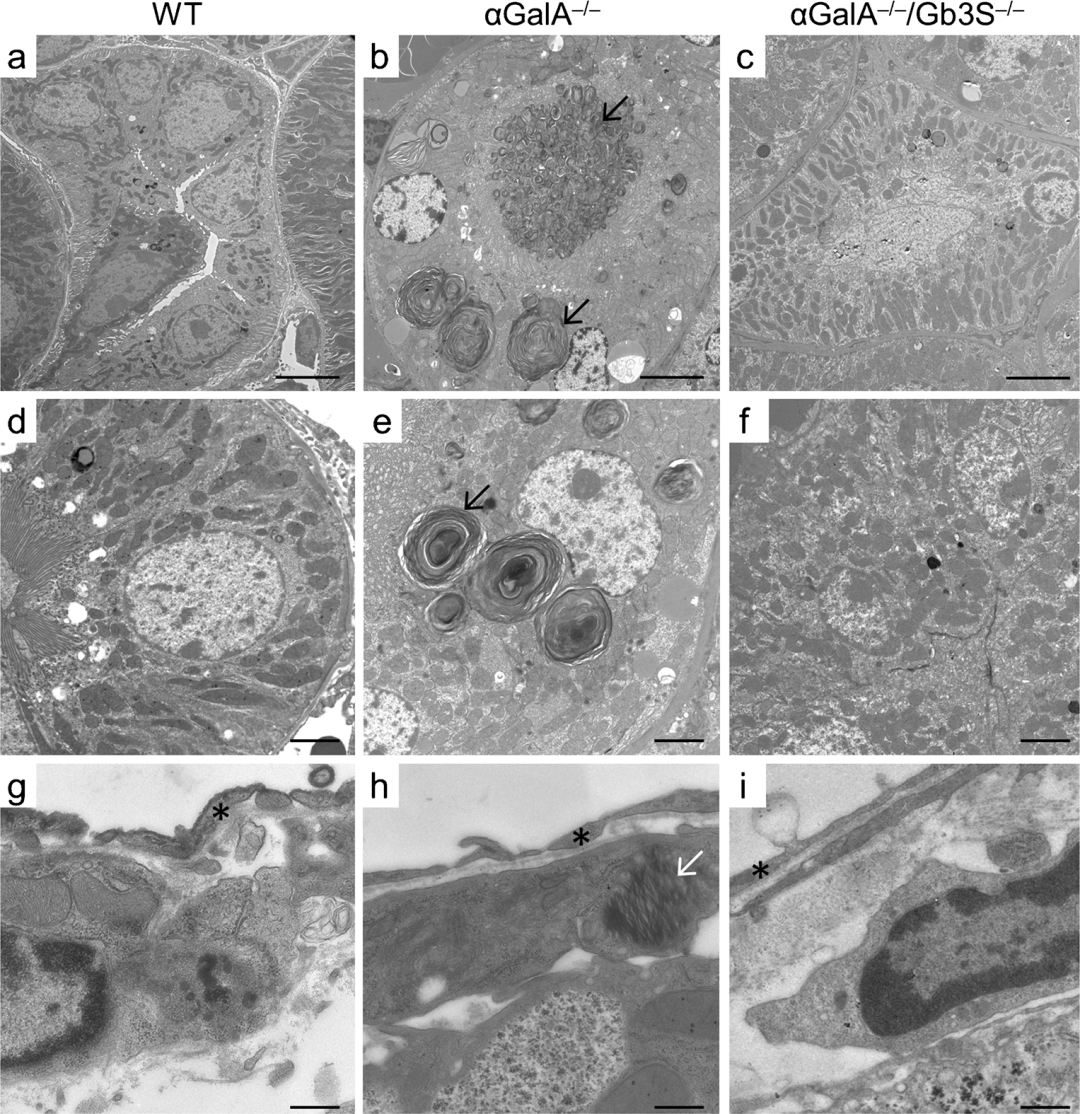
Ultrastructural analysis of selected organs. Organs of WT, α*GalA* ^*−/−*^, and α*GalA* ^*−/−*^ */Gb3S* ^*−/−*^ mice were analyzed by electron microscopy for the presence of ultrastructural correlates of a lysosomal storage disease. **a**–**f** In kidneys of α*GalA* ^*−/−*^ mice, numerous enlarged lysosomes with characteristic concentric lamellar inclusions were present in tubular epithelial cells (*black arrow*). Depletion of globosides fully restored a normal lysosomal morphology in α*GalA* ^*−/−*^ */Gb3S* ^*−/−*^ mice. **g**–**i** The storage phenotype in the liver is exemplified by Ito cells, which can be identified in the Disse’s (perisinusoid) space (*endothelial cells). αGalA deficiency resulted in a distorted lysosomal architecture (*white arrow*) in α*GalA* ^*−/−*^ mice but not in the absence of Gb3S-activity (i.e. in the α*GalA* ^*−/−*^ */Gb3S* ^*−/−*^ mice). Stain: lead citrate/uranyl acetate. *Scale bars*(**a**–**c**) 5 μm, (**d**–**f**) 2 μm, (**g**–**i**) 0.5 μm

Furthermore, organs were subjected to ultrastructural analysis, which represents the decisive method for visualization of lysosomal storage phenomena. In αGalA-deficient mice, the storage could be documented as concentric and lamellar lysosomal inclusions (Fig. 5b, e, h) which are also characteristic for the human disease. In line with the immunohistochemical findings, an extensive accumulation could be documented in renal tubular epithelial cells in αGalA-deficient mice (Fig. 5b, e). In contrast, podocytes and renal endothelial cells of αGalA-deficient mice showed a regular ultrastructure. Singular podocytes alone showed a slight lysosomal enlargement and lamellar structures resembling αGalA deficiency (data not shown).

Electron microscopy revealed the lysosomal storage also in Kupffer and Ito cells in livers of αGalA-deficient mice (Fig. 5h). In hearts of αGalA-deficient mice, endothelial and interstitial cells were affected by the storage (Electronic Supplementary Material, Fig. S2).

In α*GalA* ^*−/−*^ */Gb3S* ^*−/−*^ mice, the elimination of globosides restored a normal lysosomal morphology in kidney, liver and heart and resulted in an ultrastructural appearance indistinguishable from WT mice (Fig. 5c, f, i; Electronic Supplementary Material, Fig. S2). Thus, Gb3S deficiency was sufficient to abolish the lysosomal storage associated with αGalA deficiency in these organs, yet without causing any developmental or histological abnormalities and without interfering with organ function (Table 1 and Porubsky et al. 2007, 2012).

### Contribution of isoglobosides to the storage phenotype in dorsal root ganglia

**Fig. 6:**
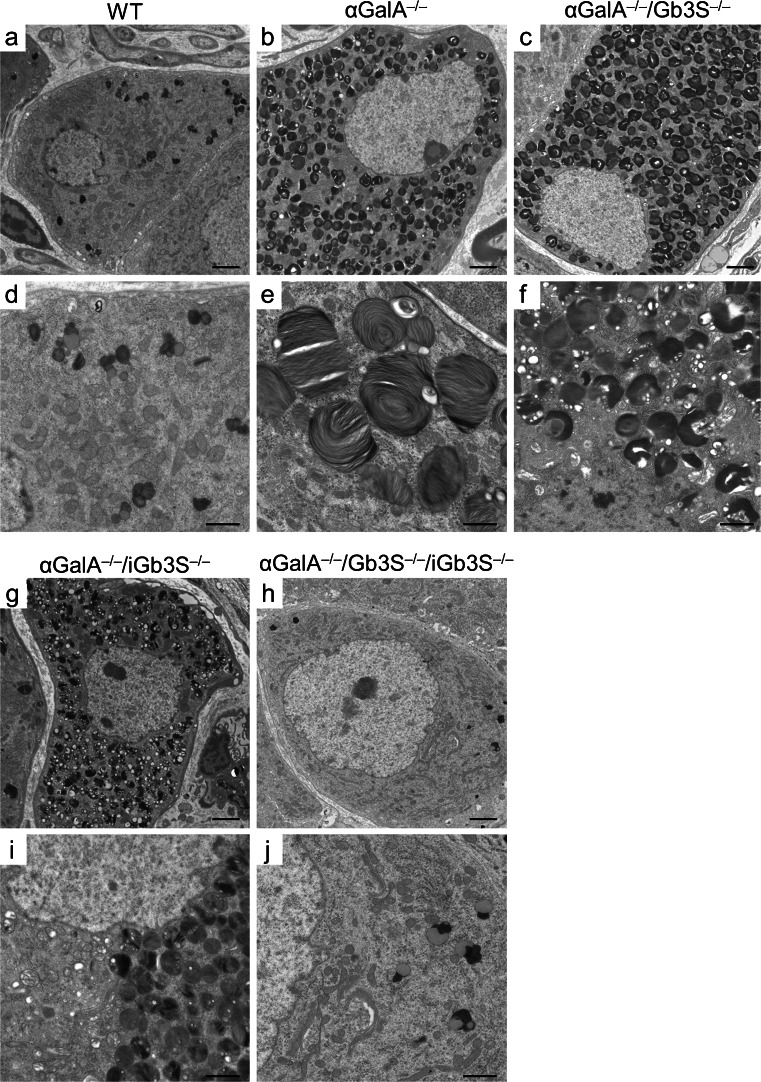
Ultrastructural analysis of dorsal root ganglia. Dorsal root ganglia were analyzed by electron microscopy. DRG of αGalA-deficient mice showed numerous enlarged lysosomes filled with lamellar material. However, in contrast to other organs, a restoration of a normal lysosomal morphology could not be achieved by depletion of globosides (α*GalA* ^*−/−*^ */Gb3S* ^*−/−*^). Similarly, depletion of isoglobosides (α*GalA* ^*−/−*^ */iGb3S* ^*−/−*^) also did not lead to a normalization of the lysosomal morphology. This could be achieved only by a simultaneous depletion of both globosides and isoglobosides (i.e. in the α*GalA* ^*−/−*^ */Gb3S* ^*−/−*^ */iGb3S* ^*−/−*^ mouse). Stain: lead citrate/uranyl acetate.* Scale bars* (**a**–**c**, **g**, **h**) 2 μm, (**d**–**f**, **i**, **j**) 1 μm

A comprehensive ultrastructural analysis of other structures revealed that—in contrast to the previously mentioned organs—in dorsal root ganglia (DRG) of α*GalA* ^*−/−*^ */Gb3S* ^*−/−*^ mice, a lysosomal storage was still present (Fig. 6). Taking into account that iGb3 is synthesized in DRG (Speak et al. 2007), we analyzed DRG of α*GalA* ^*−/−*^ */iGb3S* ^*−/−*^ mice. However, the sole deficiency in iGb3S did not suffice to counteract the storage phenotype associated with αGalA deficiency in DRG. Only a simultaneous depletion of both globosides and isoglobosides (i.e. in the α*GalA* ^*−/−*^ */Gb3* ^*−/−*^ */iGb3* ^*−/−*^ mouse) was able to abolish the lysosomal storage and restore a normal ultrastructural morphology in the DRG (Fig. 6h, j).

## Discussion

These results demonstrate that, in αGalA deficiency, depletion of globosides was sufficient to fully revert the storage phenotype in the most prominently affected organs like heart, liver, and kidney (Figs. 3, 4, 5, 6). To achieve a complete restoration of the lysosomal morphology in DRG, which also produce isoglobosides, the simultaneous depletion of both globosides and isoglobosides was necessary (Fig. 6). The latter finding is supported by a previous investigation, which documented the synthesis of iGb3 only in the DRG but not in other murine organs (Speak et al. 2007).

The murine model of Fabry disease used in this study shows—in congruence with the human disease—a complete lack of lysosomal αGalA activity, which results in an aberrant lysosomal ultrastructure and function (Ohshima et al. 1997; Ohashi et al. 2008; Porubsky et al. 2012). However, in contrast to Fabry patients, an effect on the function of heart, kidney, and brain is completely absent or only very mild, and the life span is normal in αGalA-deficient mice. In particular, no ophthalmologic manifestations (Ohshima et al. 1999) and no hearing loss (Noben-Trauth et al. 2007) could be demonstrated, although some studies have reported slight sensorimotor alterations (Rodrigues et al. 2009; Marshall et al. 2010) and a marginal myocardial affection (Yoshimitsu et al. 2006; Rozenfeld et al. 2011; Nguyen Dinh Cat et al. 2012). The latter could not be verified in our colony, possibly due to the extensive backcrossing towards the reference C57BL/6 strain and a strict breeding of all strains under identical conditions (Fig. 2). Also in line with previous investigations (Ohshima et al. 1999), we could not detect any decline in kidney function (measured as creatinine clearance) and no proteinuria or microalbuminuria in aged αGalA-deficient mice (Fig. 2). The increased kidney weight in α*GalA* ^*−/−*^ females as compared to WT, α*GalA* ^*−/−*^ */Gb3S* ^*−/−*^, and *Gb3S* ^*−/−*^ mice (Table 1) did not show any correlates upon light microscopy (data not shown). Similarly, the elevated serum cholesterol in male α*GalA* ^*–/0*^ mice (Table 1) was not reflected by any light microscopic changes in the liver or any other organ (including heart; data not shown).

The aforementioned absent or very slight functional alterations in the murine model of Fabry disease contrast with the extensive GSL accumulation and the amply documented derangement of lysosomal morphology in the organs of these mice (Figs. 3, 4, 5, 6). The paucity of functional correlates in this mouse model is probably due to the fact that the cells which determine the organ affection and morbidity in humans (e.g., podocytes in the kidney or endothelial cells in the heart and brain) do not express sufficient amounts of Gb3S in mice and are thus not affected by the storage to an extent which would allow the development of similar symptoms. This explanation is supported by a recent report which showed that αGalA-deficient mice were symptomatic when crossbred with transgenic mice ubiquitously expressing Gb3S (Taguchi et al. 2013). αGalA-deficient mice and humans show identical GSL storage patterns with globosides constituting the vast majority of the accumulating GSL (Sweeley and Klionsky 1963; Hozumi et al. 1990; Ohshima et al. 1999). Therefore, although the αGalA-deficient mouse model is not suited for functional studies, it represents a useful tool to investigate biochemical and ultrastructural alterations and possible therapeutic approaches in vivo. In our study, we did not determine the levels of lyso-Gb3, which has been postulated as a marker of Fabry disease (Aerts et al. 2008; Togawa et al. 2010), as it can be anticipated that, in the α*GalA* ^*−/−*^ */Gb3S* ^*−/−*^ mouse, the abolishment of the synthesis of Gb3 will curtail any production of lyso-Gb3 (Fig. 3).

To date, the only accepted therapy for Fabry disease is ERT. Besides its high costs and still unproven efficacy, the most prominent problem of the intravenously administered enzyme is its bioavailability in target organs. In this regard, an approach through SRT seems to be advantageous because tissue availability of small inhibitors can be optimized by molecular engineering. SRT through inhibition of GCS (Fig. 1) seems to be possible as a therapeutic option for αGalA deficiency (Abe et al. 2000; Platt et al. 2003). However, such an approach also results in depletion of other GSL groups, which do not contribute to the storage in αGalA deficiency. Moreover, GSC-inhibition does not interfere with the synthesis of galabiosylceramide in the kidney (Fig. 1) and thus cannot abolish its accumulation in Fabry disease. Although it is unclear to which extent the accumulation of galabiosylceramide itself contributes to the renal phenotype of Fabry disease, inhibition of the Gb3S would—in contrast to the inhibition of GCS—eliminate the accumulation not only of globosides but also of galabiosylceramide.

It could be demonstrated that deficiency in globosides is protective upon exposure to Shigatoxins (Okuda et al. 2006; own unpublished data); however, the physiological function of globosides and isoglobosides remains elusive as no derangements of physiological functions have so far been observed in mice deficient for *Gb3S* and/or *iGb3S* (Table 1; Electronic Supplementary Material, Fig.S1; Porubsky et al. 2007, 2012).

In summary, our study explored the possibility of a targeted SRT for Fabry disease through the depletion of globosides and isoglobosides, which both seem to be dispensable for all biological functions tested so far (Table 1; Porubsky et al. 2007, 2012). We show that interference with the Gb3S activity abolishes the GSL storage and normalizes lysosomal morphology in most organs. For a complete elimination of storage phenomena in DRG, isoglobosides have to be depleted in addition to globosides. We are convinced that a pharmacologic inhibition of Gb3S and iGb3S represents a potentially successful SRT for Fabry disease. Experiments are in progress to identify specific inhibitors of Gb3S and iGb3S.

## Electronic supplementary material

Below is the link to the electronic supplementary material.

Electronic Supplementary Material, Figure S1 (PDF 200 kb)

Electronic Supplementary Material, Figure S2 (PDF 131 kb)
