# Supplementary material for: Depletion of globosides and isoglobosides fully reverts the morphologic phenotype of Fabry disease
Source: Cell Tissue Res. 2014 Jul 4;358(1):217–27. doi: 10.1007/s00441-014-1922-9 (PMC4186980; doi:10.1007/s00441-014-1922-9)
Supplement: Supplementary file 1 — (PDF 200 kb) [file 441_2014_1922_MOESM1_ESM.pdf]

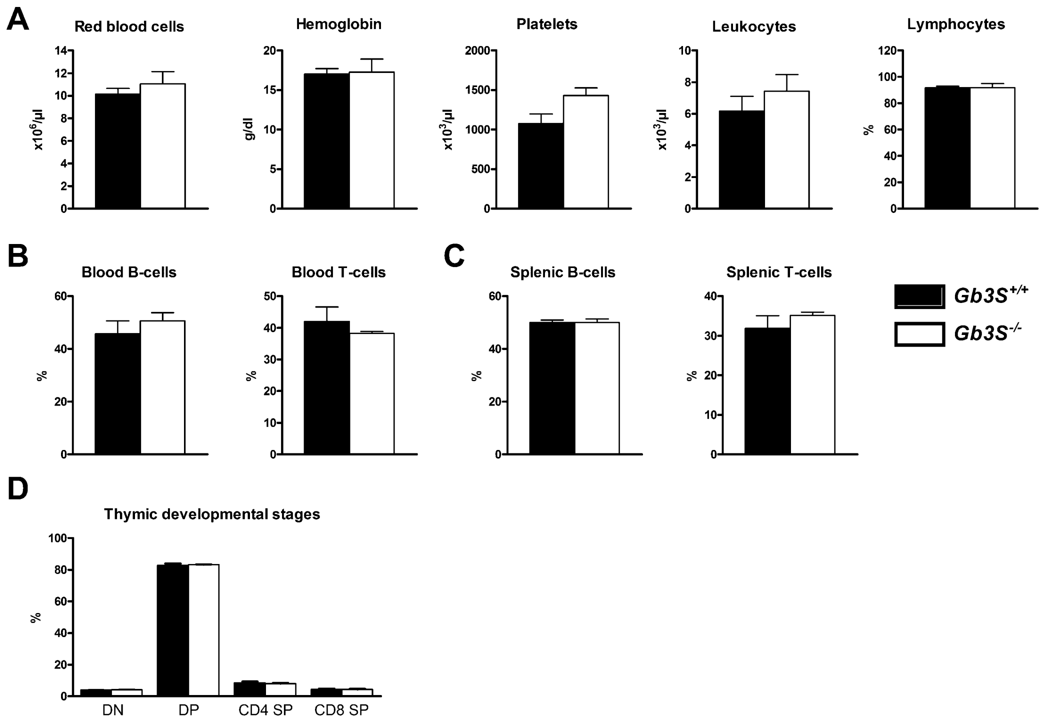

Electronic Supplementary Material, Figure S1: Analysis of peripheral blood, spleen and thymus in Gb3-deficient mice.

Peripheral blood (A-B), spleen (C) and thymus (D) were analyzed by flow cytometry. No statistically significant differences between WT and Gb3-deficient mice could be observed in any of the parameters investigated. Bars represent means, depicted are standard errors of means, n = 3-4. DN, double negative ($CD4^-/CD8^-$); DP, double positive ($CD4^+/CD8^+$); SP, single positive.
