# Supplementary material for: Depletion of globosides and isoglobosides fully reverts the morphologic phenotype of Fabry disease
Source: Cell Tissue Res. 2014 Jul 4;358(1):217–27. doi: 10.1007/s00441-014-1922-9 (PMC4186980; doi:10.1007/s00441-014-1922-9)
Supplement: Supplementary file 2 — (PDF 131 kb) [file 441_2014_1922_MOESM2_ESM.pdf]

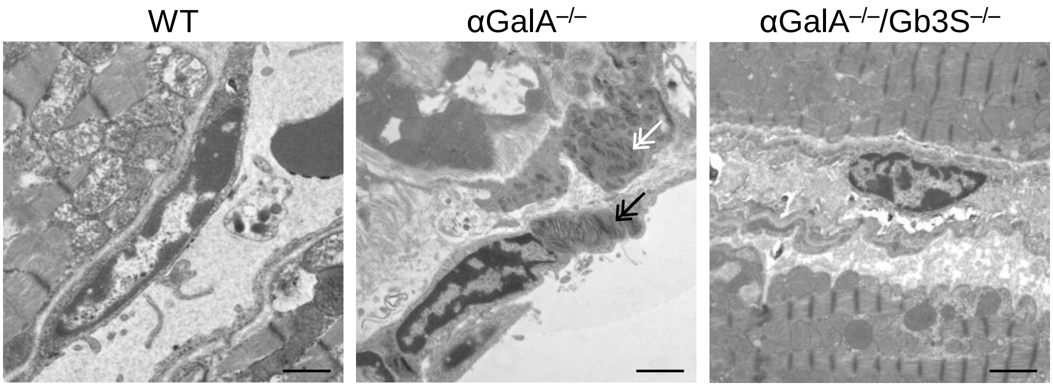

Electronic Supplementary Material, Figure S2: Ultrastructural analysis of hearts. In hearts of αGalA-deficient mice, the storage phenotype was expressed in endothelial and interstitial cells (black and white double arrow, respectively). Also here, the storage phenotype was fully dependent on the activity of Gb3S as evidenced by the αGalA$^{-/-}$/Gb3S$^{-/-}$ mice. Stain: lead citrate/uranyl acetate. Scale bars represent 2µm.
